# Association between Carotid Plaque and Cognitive Impairment in Chinese Stroke Population: The SOS-Stroke Study

**DOI:** 10.1038/s41598-017-02435-3

**Published:** 2017-06-08

**Authors:** Anxin Wang, Xiaoxue Liu, Guojuan Chen, Hongjun Hao, Yongjun Wang, Yilong Wang

**Affiliations:** 1 Department of Neurology, Beijing Tiantan Hospital, Capital Medical University, Beijing, China; 2 China National Clinical Research Center for Neurological Diseases, Beijing, China; 3 Center of Stroke, Beijing Institute for Brain Disorders, Beijing, China; 4 Beijing Key Laboratory of Translational Medicine for Cerebrovascular Disease, Beijing, China; 5 Department of Cardiology, Tangshan People’s Hospital, Tangshan, China; 6 Department of Neurology, Tangshan Gongren Hospital, North China University of Science and Technology, Tangshan, China; 7 Department of Neurology, Peking University First Hospital, Beijing, China

## Abstract

We aimed to investigate the association between carotid plaques and cognitive impairment among patients with acute ischemic stroke, and to assess key clinical implications. In the Acute Ischemic Stroke Study, patients who received a cognitive testing and underwent complete carotid artery ultrasound scans were included. Cognitive function was measured by the mini-mental state examination. The cross-sectional relationships between cognitive impairment and carotid plaques were evaluated using multivariate logistic regression analysis. Of the 3116 patients included in this study, 826 (26.51%) patients were diagnosed with cognitive impairment. After adjusting for potential confounders, patients with ≥2 carotid plaques (odds ratio [OR] = 1.47; 95% confidence interval [CI]: 1.19–1.82), patients with ≥2 number of carotid arteries with plaque (OR = 1.48; 95% CI: 1.19–1.84) and patients with hypoechoic plaque (OR = 2.05; 95% CI: 1.24–3.38) are more likely to have cognitive impairment. In this acute ischemic stroke population, the number of carotid plaques, the number of carotid arteries with plaque and plaque stability are all associated with cognitive impairment.

## Introduction

Vascular risk factors and cardiovascular diseases are associated with both vascular dementia and Alzheimer’s disease^1–5^. Western studies investigating the association between carotid atherosclerosis and cognitive impairment have been performed in elderly people^6–9^ and in younger adults^10, 11^ without prior stroke, and they have demonstrated that carotid atherosclerosis may be a risk factor for cognitive impairment. Increased carotid intima-media thickness (IMT), reflecting atherosclerosis, has been associated with an increased risk of cognitive decline^12–16^. Compared with early intima-media thickening, formed plaques represent more advanced atherosclerosis, which are close to becoming artery stenosis and thrombosis. A prospective cohort showed that plaque number was associated with increased risk of dementia and mortality^17^. However, another prospective study found no increased risk of dementia or Alzheimer’s disease in persons with atherosclerotic plaques^18^.

Elias MF et. have reported that higher 10-year Framingham risk for stroke was associated with performance decrements in multiple cognitive domains^19^. Several other studies also found that carotid plaques were a major risk factor for stroke and subsequent cognitive impairment. However, most of the above studies checking for the association between carotid plaques and cognitive impairment were conducted in stroke-free individuals^20^, and that the results of these studies are partly inconsistent. In our study, we aimed to examine the association between carotid plaques measured by carotid ultrasound and cognitive impairment during a large multi-center sample of stroke patients.

## Results

The mean age of the 3116 patients included in this study is 63.42 ± 11.87 years. Among them, 826 (26.51%) patients were diagnosed with cognitive impairment. Supplementary Table 1 shows the characteristics of patients according to the inclusion and exclusion criteria in this study. Compared with patients included (n = 3116), patients excluded (n = 1048) are older, more frequently married, have higher National Institutes of Health Stroke Scale (NIHSS) score, and have higher proportion of atrial fibrillation, large artery atherosclerosis and cardioembolism and lower prevalence of hypercholesterolemia.

Table 1 presents the characteristics of patients with good cognition as well as those with cognitive impairment. Age, sex, marital status, education, alcohol use, tobacco use, hypercholesterolemia, and atrial fibrillation are associated with cognitive impairment (p < 0.05). Compared with patients with good cognition, patients with cognitive impairment have a higher median NIHSS score, an increased proportion of large artery atherosclerosis and cardioembolism, an increased number of plaques, and an increased proportion of hypoechoic plaques.

**Table 1:** Characteristics of patients with and without cognitive impairment in the study population.

Variable	Cognitively intact (MMSE ≥ 24)	Cognitively impaired (MMSE < 24)	p value
n	2290	826	
Age, years	62.02 ± 11.72	67.19 ± 11.84	<0.0001
Sex (men)	1570 (68.56%)	461 (55.81%)	<0.0001
Married (Yes)	2157 (94.19%)	751 (90.92%)	0.0012
Alcohol use (Yes)	753 (32.88%)	207 (25.06%)	<0.0001
Tobacco use (Yes)	961 (41.97%)	258 (31.32%)	<0.0001
Physical activity (Yes)	910 (39.74%)	267 (32.32%)	0.0002
Education level, n (%)			
Middle school and below	2071 (90.44%)	751 (90.92%)	0.6837
High school and above	219 (9.56%)	75 (9.08%)	
History of disease, n (%)			
Hypertension	1506 (65.76%)	558 (67.55%)	0.3509
Diabetes	541 (23.62%)	176 (21.31%)	0.1750
Hypercholesterolemia	283 (12.36%)	76 (9.20%)	0.0148
Atrial fibrillation	88 (3.84%)	55 (6.66%)	0.0009
Coronary artery disease	54 (2.36%)	21 (2.54%)	0.7670
NIHSS, score	4 (2–6)	8 (4–12)	<0.0001
TOAST classification, n (%)			
Large artery atherosclerosis	1200 (52.40%)	533 (64.53%)	<0.0001
Cardioembolism	95 (4.15%)	62 (7.51%)	
Small vessel occlusion	913 (39.87%)	169 (20.46%)	
Other determined etiology and Undetermined etiology	82 (3.58%)	62 (7.51%)	
Plaque numbers, n (%)			
0	977 (42.66%)	313 (37.89%)	0.0024
1	583 (25.46%)	195 (23.61%)	
≥2	730 (31.88%)	318 (38.50%)	
Artery numbers with plaque, n (%)			
0	977 (42.66%)	313 (37.89%)	0.0054
1	618 (26.99%)	213 (25.79%)	
≥2	695 (30.35%)	300 (36.32%)	
Plaque echo, n (%)			
Hyperechoic	1251 (54.63%)	471 (57.02%)	0.0008
Hypoechoic	62 (2.71%)	42 (5.08%)	

MMSE: Mini-Mental State Examination; SD = Standard Deviation; NIHSS: National Institutes of Health Stroke Scale; TOAST: Trial of Org 10172 in Acute Stroke Treatment.

Table 2 shows the results of multivariate regression analyses of the association between carotid plaques and cognitive impairment. The more carotid plaques patients have, the more likely patients have cognitive impairment (p for trend = 0.0004). After adjusting for potential confounders, patients with ≥2 carotid plaques have a higher prevalence of cognitive impairment, compared with patients without carotid plaques (odds ratios [OR] = 1.47, 95% Confidence Interval [CI]: 1.19–1.82, p < 0.001).

**Table 2:** Odds ratios of cognitive impairment by number of plaques.

	Number of plaques			P for trend
	0	1	≥2	
Case number	1290	778	1048	
Model 1^*^	1	1.18 (0.95–1.46)	1.30 (1.08–1.56)	0.0063
Model 2^†^	1	1.15 (0.93–1.43)	1.31 (1.08–1.58)	0.0054
Model 3^‡^	1	1.32 (1.03–1.68)	1.47 (1.19–1.82)	0.0004

*Adjusted for age, sex. ^†^Adjusted for as model 1 plus education level, marriage status, alcohol use, tobacco use, physical activity, hypertension, diabetes, hypercholesterolemia, atrial fibrillation, coronary artery disease. ^‡^Adjusted for as model 2 plus NIHSS and TOAST. NIHSS: National Institutes of Health Stroke Scale; TOAST: Trial of Org 10172 in Acute Stroke Treatment.

The association between the number of carotid arteries with a plaque and cognitive impairment is summarized in Table 3. The more carotid arteries with plaque, the more likely patients have cognitive impairment (p for trend = 0.0004). After adjusting for all potential confounders, patients with ≥2 carotid arteries with plaque have a greater likelihood of having cognitive impairment compared with patients without carotid plaques (OR = 1.48, 95% CI: 1.19–1.84, p < 0.001).

**Table 3:** Odds ratios of cognitive impairment by number of carotid arteries with plaque.

	Number of Arteries with Plaque			P for trend
	0	1	≥2	
Case number	1290	831	995	
Model 1*	1	1.20 (0.98–1.48)	1.28 (1.06–1.55)	0.0091
Model 2^†^	1	1.18 (0.95–1.45)	1.30 (1.07–1.57)	0.0072
Model 3^‡^	1	1.31 (1.04–1.67)	1.48 (1.19–1.84)	0.0004

*Adjusted for age, sex. ^†^Adjusted for as model 1 plus education level, marriage status, alcohol use, tobacco use, physical activity, hypertension, diabetes, hypercholesterolemia, atrial fibrillation and coronary artery disease. ^‡^Adjusted for as model 2 plus NIHSS and TOAST. NIHSS: National Institutes of Health Stroke Scale; TOAST: Trial of Org 10172 in Acute Stroke Treatment.

A multivariate regression analysis is performed to investigate the association between different ultrasonic characters of carotid plaques and cognitive impairment (Table 4). Compared with patients without carotid plaque, patients with hyperechoic carotid plaques (OR = 1.38, 95% CI: 1.13–1.67, p < 0.001), and hypoechoic carotid plaques (OR = 2.05, 95% CI: 1.24–3.38, p < 0.001) are more likely to have cognitive impairment, after adjusting for all potential confounders. Additionally, patients with hypoechoic carotid plaques have almost double the risk of cognitive impairment than patients with hyperechoic carotid plaques.

**Table 4:** Odds ratios of cognitive impairment by echo character of plaques.

	Echo of plaques		
	Without Plaque	Hyperechoic	Hypoechoic
Case number	1290	1722	104
Model 1*	1	1.20 (1.01–1.42)	2.21 (1.45–3.38)
Model 2^†^	1	1.20 (1.01–1.42)	2.29 (1.49–3.51)
Model 3^‡^	1	1.38 (1.13–1.67)	2.05 (1.24–3.38)

*Adjusted for age, sex. ^†^Adjusted for as model 1 plus education level, marriage status, alcohol use, tobacco use, physical activity, hypertension, diabetes, hypercholesterolemia, atrial fibrillation and coronary artery disease. ^‡^Adjusted for as model 2 plus NIHSS and TOAST. NIHSS: National Institutes of Health Stroke Scale; TOAST: Trial of Org 10172 in Acute Stroke Treatment.

The association of different cognitive domains in the mini-mental state examination (MMSE) and carotid plaques was presented in Supplementary Table 2. Compared with patients without carotid plaque, patients with ≥2 carotid plaques, patients with ≥2 carotid arteries with plaque and patients with hypoechoic plaques have a greater likelihood to experience decreasing in orientation, registration, recall, language, attention and calculation.

## Discussion

Our study explored the association between carotid plaques and cognitive impairment in patients with acute ischemic stroke (AIS) in China. We observed a positive association between carotid plaques and cognitive impairment in the AIS patients. The main results of this study were that the number of carotid plaques, the number of carotid arteries with plaque, and the ultrasonic character of plaques were all significantly associated with cognitive impairment after adjusting potential confounders.

In the present study, increased numbers of carotid plaques were correlated with cognitive impairment in stroke patients older than 18 years. Our findings of an association between carotid plaques and risk for cognitive impairment are in line with previous reports regarding non-stroke patients^9, 11, 17, 21, 22^. This association was independent of known confounding factors, such as age, sex, education level, marital status, alcohol use, tobacco use, physical activity, hypertension, diabetes, hypercholesterolemia, atrial fibrillation, coronary artery disease, and NIHSS. Our data supports the previously published results of the Rotterdam study^9^, which found that the number of carotid plaques was associated with dementia in cross-sectional analysis^9^. In a prospective study of 4,371 stroke-free middle-aged patients, a similar association was found between mean carotid plaques and cognitive decline after a 7-year follow-up^22^. However, a significant negative trend was found between the number of carotid plaques and a combined endpoint of dementia and mortality^17^, suggesting that a higher mortality rate in subjects with carotid plaques may have attenuated a possible association between plaque number and dementia.

Previous studies have indicated that total plaque burden may be a sensitive tool for predicting clinical disease associated with atherosclerosis^23^. In our study, we did not collect the data for measurement information of total plaque burden. However, we further analyzed the association between cognitive impairment and the number of carotid arteries with plaque, which may reflect the total plaque burden to a certain extent. After adjusting for potential confounders, patients with higher numbers of carotid arteries with plaque had a greater likelihood to have cognitive impairment. Regardless of plaque numbers, the number of arteries with plaque is also a marker of atherosclerosis burden, leading to cognitive impairment.

The characters of carotid plaques may play an important role in mediating cognitive impairment. Therefore, we investigated the association between different plaque ultrasonic characteristics and cognitive impairment. After adjusting for risk factors of vascular diseases, patients with carotid plaques, especially hypoechoic plaques, had a greater likelihood of having cognitive impairment compared with patients without carotid plaques. This finding is consistent with another Chinese study^24^ conducted in a stroke-free population. In their study of 2,015 participants aged 65–85 years, an association between soft carotid plaques (hypoechoic plaques) and cognitive decline was found. This confirmed the finding that unstable carotid plaques assessed by hypoechoic imaging are also a marker for underlying high-risk factors, and generalized atherosclerosis for cognitive impairment.

Several mechanisms may explain the relationship between subclinical carotid atherosclerosis assessed by carotid plaques and cognitive impairment. First, atherosclerosis may cause hypoperfusion, which may in turn cause brain dysfunction^25^. Second, atherosclerosis may be a marker for other pathogenic pathways, such as inflammation and endothelial dysfunction, and these pathways may contribute to atrophy in the brain leading to cognitive decline^26, 27^. Third, atherosclerosis, especially unstable carotid plaques, may cause cerebral emboli, and thus cause cognitive impairment^28^. Additionally, the increased variability in blood pressure among AIS patients could be implicated in the pathogenesis of cognitive impairment^29–31^. Studies have shown associations of variability in blood pressure with atherosclerosis, leukoaraiosis, and cognitive decline after years.

An important strength of our study was the multi-center design, which was based on a consecutively selected population of AIS patients in 43 hospitals across China. However, a potential limitation of this study is that measures of atherosclerosis were not available in all patients. In addition, these hospitals in our study were not randomly selected from the whole hospitals in China. Therefore, our results may not be generalizable to the all stroke patients in China. And this study was a cross-sectional study, which limits our ability to assess a cause-effect relationship between carotid plaques and cognitive impairment. Finally, we didn’t collect the information of the time from stroke onset to discharge and the size, location, and vascular distribution of infarction, which could bias the results.

## Conclusions

In conclusion, the number of carotid plaques, the number of carotid arteries with plaque and plaque stability were all associated with cognitive impairment in patients with AIS in China. The hypothesis deriving from the above results that intervention for plaque regression may delay the cognitive impairment should be investigated clinically in the future.

## Methods

### Study Design and Population

The Study on Oxidative Stress in Patients with Acute Ischemic Stroke (SOS-Stroke) was a prospective, multi-center registry, which enrolled consecutively selected patients with AIS. There were 4164 patients admitted to 43 designated hospitals in China from January 2014 to October 2014. The inclusion and exclusion criteria for the SOS-Stroke have been published previously^32, 33^. Briefly, patients who aged over 18 years and had been diagnosed with AIS within 14 days were included. Out of the 4164 patients, we excluded 417 patients with incomplete data from MMSE, and 631 patients with incomplete data for carotid plaques. Ultimately, 3116 patients, of whom 2031 (65.18%) were men, were included in data analysis for this study. The SOS-Stroke study was sponsored by the Stroke Screening and Prevention Engineering Office of the National Health and Family Planning Commission, and was approved by the Ethics Committee of Beijing Tiantan Hospital, Xuanwu Hospital Capital Medical University, and Peking Union Medical College Hospital, in compliance with the Declaration of Helsinki. All patients or their legal authorized representatives signed informed consent before participation.

### Ultrasound Examination

Carotid atherosclerosis was assessed by experienced local investigators with high-resolution B-mode ultrasonography with a 7.5 MHz probe, based on a slight modification of the Atherosclerosis Risk in Communities protocol^34, 35^. Carotid plaques were determined at 6 different locations: common carotid artery, carotid bifurcation, and internal carotid artery on both the left and right sides^36^. The atherosclerotic plaque was defined as a focal structure encroaching into the arterial lumen of at least 0.5 mm, or 50% of the surrounding IMT value, or a thickness >1.5 mm as measured from the media-adventitia interface to the intima-lumen interface^37^. The plaque stability was determined based on the plaque echodensity. In this study, an unstable plaque was defined as a hypoechoic plaque, while a stable plaque was defined as a hyperechoic plaque. The examining results were then reviewed by two other independent operators. Discrepancies between their evaluations were resolved by consensus.

### Neuropsychological Evaluation

We used the MMSE to measure patients’ cognitive function. The MMSE is a general cognitive measure including orientation to time and place, attention and calculation, language, and memory^38^. Higher scores indicate greater cognitive function. A score of less than 24 out of 30 is defined as cognitive impairment. The MMSE in our study was performed before patients’ discharge.

### Assessment of Potential Covariates

Information regarding demographic and clinical characteristics (age, sex, marital status, alcohol use, tobacco use, physical activity, education level, and history of diseases) was collected via questionnaires. Marital status was stratified as married or unmarried (including single, divorced, or widowed). Alcohol use was defined as a daily intake of at least 100 ml of liquor 3 times per week for more than 1 year. Physical activity was evaluated regarding the type and frequency of physical activity at work and during leisure time. Previous history of disease, including hypertension, diabetes, hypercholesterolemia, atrial fibrillation, and coronary artery disease, was determined via a self-report. The score of NIHSS was recorded by a stroke neurologist on the first day of admission.

### Statistical Analyses

Statistical analyses are performed using a commercially available software program (SAS software, version 9.4; SAS Institute Inc., Cary, NC, USA). Data are presented as mean ± SD for continuous variables, and frequencies and percentages for categorical variables. We use either Student’s t-test to compare normally distributed variables, or the Wilcoxon test to compare non-parametric variables. The Chi-squared test is applied to compare categorical variables. Third, the entire study population is divided into 3 groups according to number of plaques: group 1 (0 plaque), group 2 (1 plaque), and group 3 (≥2 plaques). Variables are compared between the 3 groups. Multivariate OR are then obtained via multivariate logistic regression analysis after adjusting for possible confounders, including age, sex, education level, marital status, alcohol use, tobacco use, physical activity, hypertension, diabetes, hypercholesterolemia, atrial fibrillation, coronary artery disease, and NIHSS score. A p-value less than 0.05 (2-sided) is considered significant.

## Electronic supplementary material

Supplementary Tables
