# Supplementary material for: Association between Carotid Plaque and Cognitive Impairment in Chinese Stroke Population: The SOS-Stroke Study
Source: Sci Rep. 2017 Jun 8;7:3066. doi: 10.1038/s41598-017-02435-3 (PMC5465192; doi:10.1038/s41598-017-02435-3)
Supplement: Supplementary file 1 — Supplementary Tables [file 41598_2017_2435_MOESM1_ESM.pdf]

Full title: Association between Carotid Plaque and Cognitive Impairment in Chinese Stroke Population: The SOS-Stroke Study

Running title: Carotid Plaque and Cognitive Impairment

Authors: Anxin Wang, PhD[1,2,3,4]; Xiaoxue Liu, MD[5]; Guojuan Chen, MD[6]; Hongjun Hao, MD[7]; Yongjun Wang, MD[1,2,3,4#]; Yilong Wang, MD, PhD[1,2,3,4#].

Author's Affiliation:

1. Department of Neurology, Beijing Tiantan Hospital, Capital Medical University, Beijing, China.
2. China National Clinical Research Center for Neurological Diseases, Beijing, China.
3. Center of Stroke, Beijing Institute for Brain Disorders, Beijing, China.
4. Beijing Key Laboratory of Translational Medicine for Cerebrovascular Disease, Beijing, China.
5. Department of Cardiology, Tangshan People's Hospital, Tangshan, China.
6. Department of Neurology, Tangshan Gongren Hospital, North China University of Science and Technology, Tangshan, China.
7. Department of Neurology, Peking University First Hospital, Beijing, China.

#Correspondence to:

Yilong Wang, MD, PhD

Address: No.6 Tiantanxili, Dongcheng District, Beijing, China, 100050.

Email: yilong528@gmail.com.

Fax: +86-10-67098351.

Tel: +86-10-67098222.

Yongjun Wang, MD

Address: Department of Neurology, Beijing TianTan Hospital, Capital Medical University, No 6 Tiantanxili, Dongcheng District, Beijing 100050, China.

Email: yongjunwang1962@gmail.com

Fax: +86-010-67013383

Tel: +86-010-67098351

Supplementary Tables

Supplementary Table 1. Characteristics of patients according to the inclusion and exclusion.

Variable	Patients included n = 3116	Patients excluded n = 1048	p value
Age ,years	63.42 ± 11.87	65.45 ± 12.60	＜0.0001
Sex (men)	2031 (65.18%)	671 (64.03%)	0.4987
Married (Yes)	2908 (93.32%)	999 (95.32%)	0.0200
Alcohol use (Yes)	960 (30.81%)	302 (28.82%)	0.2248
Tobacco use (Yes)	1219 (39.12%)	382 (36.45%)	0.1243
Physical activity (Yes)	1177 (37.77%)	372 (35.50%)	0.1872
Education level, n (%)			
Middle school and below	2822 (90.56%)	951 (90.74)	0.8632
High school and above	294 (9.44%)	97 (9.26%)	
History of disease, n (%)			
Hypertension	2064 (66.24%)	678 (64.69%)	0.3618
Diabetes	717 (23.01%)	255 (24.33%)	0.3815
Hypercholesterolemia	359 (11.52%)	84 (8.02%)	0.0015
Atrial fibrillation	143 (4.59%)	98 (9.35%)	<0.0001
Coronary artery disease	75 (2.41%)	22 (2.10%)	0.5678
NIHSS, score	4 (2-7)	5 (2-11)	＜0.0001
TOAST classification, n (%)			

Large artery atherosclerosis	1733 (55.62%)	653 (62.31%)	＜0.0001
Cardioembolism	157 (5.04%)	103 (9.83%)	
Small vessel occlusion	1082 (34.72%)	229 (21.85%)	
Other determined etiology and Undetermined etiology	144 (4.62%)	63 (6.01%)	

SD: Standard Deviation; MMSE: Mini-Mental State Examination; NIHSS: National Institutes of Health Stroke Scale; TOAST: Trial of Org 10172 in Acute Stroke Treatment.

Supplementary Table 2. Odds ratios of different cognitive domains in MMSE by carotid plaques *.

MMSE	Plaque numbers		Artery numbers with plaque		Plaque echo	
	1[#]	⩾2[#]	1[#]	⩾2[#]	Hyperechoic[#]	Hypoechoic[#]
Orientation	1.30 (1.05-1.60)	1.53 (1.28-1.84)	1.29 (1.05-1.58)	1.56 (1.29-1.87)	1.42 (1.20-1.68)	1.67 (1.08-2.57)
Registration	1.30 (1.03-1.65)	1.39 (1.13-1.71)	1.25 (0.99-1.58)	1.44 (1.17-1.78)	1.34 (1.12-1.62)	1.67 (1.04-2.66)
Attention and Calculation	1.45 (1.21-1.73)	1.49 (1.27-1.74)	1.42 (1.20-1.69)	1.51 (1.29-1.77)	1.46 (1.27-1.68)	1.76 (1.20-2.58)
Recall	1.39 (1.16-1.68)	1.48 (1.26-1.75)	1.42 (1.19-1.71)	1.47 (1.24-1.73)	1.42 (1.23-1.64)	1.99 (1.35-2.94)
Language	1.25 (1.05-1.50)	1.39 (1.18-1.62)	1.25 (1.05-1.49)	1.40 (1.19-1.64)	1.30 (1.13-1.50)	2.03 (1.39-3.00)

* Adjusted for as age, sex, education level, marriage status, alcohol use, tobacco use, physical activity, hypertension, diabetes, hypercholesterolemia, atrial fibrillation, coronary artery disease, NIHSS and TOAST.

[#] Refer to the group without carotid plaque.

MMSE: Mini-Mental State Examination; NIHSS: National Institutes of Health Stroke Scale; TOAST: Trial of Org 10172 in Acute Stroke Treatment.
